# Strategic acquisition of healthcare equipment in Brazil: balancing legislative amendments and research funding agencies

**DOI:** 10.1016/j.clinsp.2025.100840

**Published:** 2025-11-19

**Authors:** Everson L.A. Artifon, Marcio Roberto Facanali, José Maria Soares, Edmund Chada Baracat

**Affiliations:** a Departamento de Cirurgia, Hospital das Clínicas da Faculdade de Medicina da Universidade de São Paulo (HCFMUSP), São Paulo, SP, Brazil; b Departamento de Gastroenterologia, Hospital das Clínicas da Faculdade de Medicina da Universidade de São Paulo (HCFMUSP), São Paulo, SP, Brazil; c Departamento de Obstetrícia e Ginecologia, Hospital das Clínicas da Faculdade de Medicina da Universidade de São Paulo (HCFMUSP), São Paulo, SP, Brazil

## Editorial

The acquisition of healthcare equipment represents a crucial strategic element for institutional sustainability and technological advancement within the Brazilian Unified Health System (SUS). This editorial analyzes the public funding mechanisms that enable such acquisitions, focusing on the intersection between legislative amendments and research funding agencies as complementary tools for promoting innovation and equity in public health. The article aims to clarify the regulatory and administrative landscape that governs capital investment in healthcare, providing a conceptual framework for decision-makers involved in resource mobilization.

## Conceptual and methodological context

The reflections presented here derive from more than eight years of institutional experience in health resource mobilization, complemented by policy analysis based on public databases such as SIGEM (National System for the Management of Equipment and Materials) and RENEM (National Registry of Equipment and Permanent Materials Eligible for SUS Funding), as well as funding reports from FINEP and FAPESP. This approach provides an applied and evidence-based perspective on how to balance political opportunities with structured, long-term financing mechanisms.

Two main categories of legislative amendments deserve mention: operational and investment amendments. Operational amendments (type 2E90) are easier to release, generally within six months, and allow for greater functional flexibility in resource allocation. In contrast, investment amendments (type 8535) involve more complex bureaucratic processes at the federal level, requiring longer timelines, often two to three years, from project conception and parliamentary negotiation to final equipment delivery.^1^

Another relevant aspect is the necessity for equipment to be registered within the National System for the Management of Equipment and Materials (SIGEM) and included in the National Registry of Equipment and Permanent Materials Eligible for SUS Funding (RENEM), both maintained by the Brazilian Ministry of Health. These systems regulate and authorize the acquisition of medical devices and hospital equipment purchased with federal public resources. A notable case concerns robotic surgical platforms, which currently lack RENEM registration and therefore cannot be purchased through parliamentary amendments, although acquisition remains feasible via research funding agencies.^1^^,^^3^

Brazil hosts a wide range of agencies that support innovation, research, and technological development, including the Financier of Studies and Projects (FINEP), National Council for Scientific and Technological Development (CNPq), Brazilian Micro and Small Business Support Service (SEBRAE), Brazilian Company of Industrial Research and Innovation (EMBRAPII), Department of Science and Technology of the National Health Fund (DECIT-FNS), Coordination for the Improvement of Higher Education Personnel (CAPES), and the State Research Support Foundations (FAPs) across all federative units.^2^^,^^3^

These agencies provide highly competitive but more agile funding alternatives compared with legislative amendments. In 2024, Brazilian federal parliamentary amendments reached approximately R$85 billion (US$15 billion), while funding allocated to reimbursable innovation projects totaled R$10.7 billion (US$1.9 billion), and new financing contracts surpassed R$15 billion (US$2.7 billion), amounting to R$25.7 billion (US$4.6 billion) overall. At the state level, São Paulo’s research foundation (FAPESP) recorded a historic milestone, with revenues of R$2.8 billion (US$0.5 billion) allocated to more than 27,000 grants and projects, an 18 % increase over the previous year, marking the highest investment in the past five years.^2^^,^^3^

Based on this experience, we recommend that healthcare institutions seek operational funding through federal and state legislative amendments while directing investment demands toward national and state funding agencies. This dual strategy ensures greater efficiency, sustainability, and alignment with long-term public health goals. Ultimately, all resource mobilization initiatives should be oriented toward the institutionalization of equitable public policies that strengthen the Brazilian Unified Health System (SUS) and promote patient-centered innovation.

## Data availability statement

The datasets generated and/or analyzed during the current study are available from the corresponding author upon reasonable request.

## Declaration of competing interest

The authors declare no conflicts of interest.
